# Multifactorial Regulation Mechanisms of Negative Differential Resistance in Macropores

**DOI:** 10.3390/molecules31172962

**Published:** 2026-08-25

**Authors:** Long Ma, Haifeng Liang, Xuanji Jia, Shengjie Zhao, Jie Cheng, Hongwen Zhang

**Affiliations:** 1 School of Mechanical Engineering, Northeast Electric Power University, Jilin 132012, China; long.ma@neepu.edu.cn (L.M.); lianghaifeng000404@163.com (H.L.); zsj2831172951@163.com (S.Z.); 2 School of Energy and Power Engineering, Northeast Electric Power University, Jilin 132012, China; 17347968974@163.com; 3 School of Mechanical Engineering, Shandong University, Jinan 250061, China

**Keywords:** negative differential resistance, ion transport, cylindrical macropores, concentration gradient, surface charge, pore geometry

## Abstract

The negative differential resistance (NDR) effect provides nonlinear control over ionic current and has important potential in ion sensing and information storage. A multiphys-ics numerical model is established using COMSOL Multiphysics 6.3, coupling the Poisson−Nernst−Planck and Navier−Stokes equations to investigate the effects of solution concentration gradient, pore length, pore diameter, and surface charge density on NDR effect. The results indicate that the NDR effect occurs only in the negative voltage range, where concentration gradient diffusion competes with electric field driven migration. The characteristic voltage window stabilizes between −0.2 V and −0.5 V, and the total current reaches a local extremum near −0.2 V. Electromigration dominates in this range and sup-presses Cl^−^ ion diffusion, while K^+^ transport is less affected, resulting in decreased total ionic current. Under baseline conditions, the total current decreases by 26.19%, from −0.42 nA to −0.31 nA. Increasing the concentration gradient, shortening the pore length, enlarging the pore diameter, and reducing the surface charge density enhance local vortices or maintain Cl^−^ diffusion pathways, thereby strengthening NDR characteristics. This study reveals the regulation mechanisms of NDR effect by solution conditions, macropore structures, and surface properties, providing theoretical guidance for tunable ionic current devices.

## 1. Introduction

Ion transport in macropores forms the basis of research on nanofluidics, membrane separation, osmotic energy conversion, biosensing, and ionic information devices [1,2,3]. The decrease in ionic current through a nanopore with increasing voltage magnitude is known as the negative differential resistance (NDR) effect [4,5]. The NDR effect breaks the traditional linear current response, enabling complex and precise dynamic modulation of weak current signals. Liquid−phase NDR is an important nonlinear conduction mechanism that can reproduce the nonlinear signal response of biological macromolecular channels under specific conditions. It also provides a basis for designing fluidic sensors and devices for information storage and logic operations [6,7,8]. Furthermore, the solution concentration gradient, pore geometry, and surface charge density can be jointly tuned to effectively control ion selectivity, ion diffusion, and interfacial charge distribution in nanofluidic systems, leading to improved osmotic energy conversion efficiency [9,10,11]. The synergistic effect of concentration gradient−driven ion migration and electroosmotic flow modulates the local ionic environment and molecular−nanopore interactions, facilitating target analyte capture and controlled translocation near nanopore entrances. This process enhances molecular translocation−induced ionic current signals and provides a strategy for developing high−sensitivity label−free nanopore biosensing platforms [12,13,14]. Therefore, uncovering ion transport mechanisms in charged pores and developing effective regulation strategies are critical for advancing nanofluidic devices toward improved performance in osmotic energy conversion, sensitive biosensing, and information storage.

In recent years, liquid−phase NDR and its underlying nonlinear ion transport mechanisms have been widely studied in terms of concentration gradients, electroosmosis flow, and pressure driven transport [15,16,17]. Yang et al. [18] controlled the NDR effect in a single conical nanopore containing a concentration gradient and ion flux by varying the applied voltage waveform, which regulated the competition between electroosmotic flow and concentration polarization. Luo et al. [19] introduced an aqueous KCl solution and a 5 mM KCl solution in a dimethyl sulfoxide/water mixture on the opposite sides of a conical glass nanopore. A pressure difference and an external voltage were then applied across the nanopore. The resulting competition between electroosmotic flow and pressure−driven flow shifted the interface near the pore opening and induced a clear NDR response. Tan et al. [20] described the transport of the ionic liquid [Bmim][PF_6_] through narrow graphene pores using molecular dynamics simulations. An electric field gradient at the pore entrance generates hydrostatic pressure on polarized water molecules. This force blocks ions from entering the channel, and the ionic current decreases with an increase in voltage. Rabinowitz et al. [21] showed that concentration gradients induce nanoscale local fluid vortices and nonlinear electroosmotic flow near nanopore entrances, which strongly influence ion dynamics and nonlinear impedance behavior. The interplay among the flow, concentration, and electric fields induces local ion redistribution and constitutes an important physical mechanism for liquid−phase NDR [22,23].

In addition to the competition between pressure−driven and electroosmotic flows, local chemical reactions, ion specificity, and pore wall properties can also induce or modulate the NDR effect [24,25,26]. Ramirez et al. [27] regulate the local precipitation of two soluble salts near the tip of a conical nanopore by applying an external voltage. The resulting tunable NDR system exhibits an on/off ratio of 50–200 and detects Ca^2+^ concentrations ranging from 1 to 1000 mM through changes in the threshold voltage. Perez-Grau et al. [28] report that fluoride−induced NDR appears only in negatively charged channels. The effect becomes weaker at higher salt concentrations or with larger pore diameters, while a higher voltage scan rate further obscures the NDR characteristics. Yadav et al. [29] demonstrate through COMSOL Multiphysics simulations that electroosmotic flow leads to local concentration redistribution and decreased ionic conductivity in micro− to millimeter−scale channels under a salinity gradient. These effects contribute significantly to the occurrence of NDR in large−diameter channels. Lin et al. [30] investigate a pH−responsive conical mesopore in a KCl solution without an applied pressure difference. The NDR effect occurs only when the reservoir outside the pore tip contains a more conductive electrolyte, and the pore wall is sufficiently negatively charged at high pH. In addition, the surface charges inside and outside the pore and the ionic valence affect ion enrichment and depletion, electric field distribution, and electroosmotic flow intensity. These effects further alter ion transport and regulate the NDR response [31,32,33].

The ion transport mechanism strongly depends on the relationship between the pore size and the Debye length. At pore diameters close to or below the Debye length, the electrical double layers at the pore walls exhibit significant overlap. The resulting surface charge effects span the entire macropore, impart pronounced ion selectivity, and induce nonlinear ion transport. For pore diameters much larger than the Debye length, the electrical double layer is mainly confined to the near−wall region, while transport in the pore center gradually exhibits more pronounced bulk−like characteristics. However, surface effects and nonlinear ion transport do not disappear completely. Lin et al. [34] report that concentration polarization produces distinct voltage−dependent ion enrichment and depletion in pores on the scale of hundreds of nanometers, which subsequently alters the local electric field and ionic current. Peng et al. [35] and Gao et al. [36] further demonstrate that, even for a pore diameter of approximately 400 nm, which is much larger than the Debye length, a high surface charge density can induce a pronounced nonlinear ion transport response by regulating the near−wall ion distribution, concentration polarization, and electroosmotic flow. Hung et al. [37] further report that the mesoporous structure exhibits strong cation selectivity at a high negative surface charge density. Positive and negative biases generate ion−depletion and ion−enrichment regions, respectively, leading to pronounced asymmetric ionic rectification. These results indicate that ion transport in macropores is not governed by a single mechanism but is jointly affected by diffusion, electromigration, and concentration polarization at the pore entrance. These coupled effects alter the intrapore ion distribution and local transport resistance, providing an important physical basis for liquid−phase NDR.

Current research primarily explores how pressure−driven flow, electroosmotic flow, pore structure, and solution concentration govern the formation and regulation of NDR. Luo et al. [38] use solid state conical nanopore experiments and finite−element simulations to study ion transport driven by both a pressure difference and a KCl concentration gradient. They demonstrate that positive feedback between ionic distributions and electroosmotic flow allows the pore to transition from high to low conductance with a voltage change below 2 mV. Laucirica et al. [39] explore the mechanism of ion transport in bullet-shaped solid−state nanopores filled with KClO_4_ solutions. Under a forward voltage, ion enrichment inside the pore raises the local ion product above the solubility product, resulting in salt precipitation and NDR behavior. In contrast, no comparable response is detected in cylindrical or cigar−shaped channels. Cervera et al. [40] study the multiple NDR phenomena in charged conical nanopores based on a Boltzmann−type conductance distribution model, revealing that salt precipitation near the pore entrance at different voltage thresholds leads to consecutive decreases in ionic current. However, existing studies mainly rely on specialized pore geometries or additional regulation mechanisms. For regular cylindrical macropores, how key parameters such as the concentration gradient, pore length, pore diameter, and surface charge density regulate pore−mouth concentration polarization, local hydrodynamic behavior, and cation/anion selective transport, thereby influencing the peak−valley features and voltage ranges of NDR responses, remains to be fully understood. This study establishes a negatively charged cylindrical macropore model using COMSOL Multiphysics. Ion transport within the macropore is systematically described by coupling the Poisson−Nernst−Planck and Navier−Stokes equations. A unified numerical framework is used to systematically explore the effects of the concentration gradient, pore length, pore diameter, and surface charge density on the NDR effect in macropores. The total ionic current, individual K^+^ and Cl^−^ currents, ion concentration distributions, and local fluid velocity are analyzed to reveal the regulatory mechanism of each parameter. The results indicate that NDR is restricted to the negative-voltage region in which the concentration gradient opposes the electric field. Under all examined conditions, the characteristic voltage window remains between −0.5 and −0.2 V. Within the characteristic voltage range, the diffusive transport of Cl^−^ ions is significantly suppressed. The Cl^−^ ions current decreases rapidly and dominates the decline in the total ionic current. An increased concentration gradient, a larger pore diameter, a shorter pore length, and a lower surface charge density favor the maintenance of the Cl^−^ current and strengthen the peak and valley features of NDR effect. This study systematically relates various factors to the NDR effect in terms of solution conditions, pore architecture, and surface charge, providing theoretical support for the control of ionic transport through macropores and the design of tunable ion−current devices.

## 2. Simulation Details

**Figure 1:**
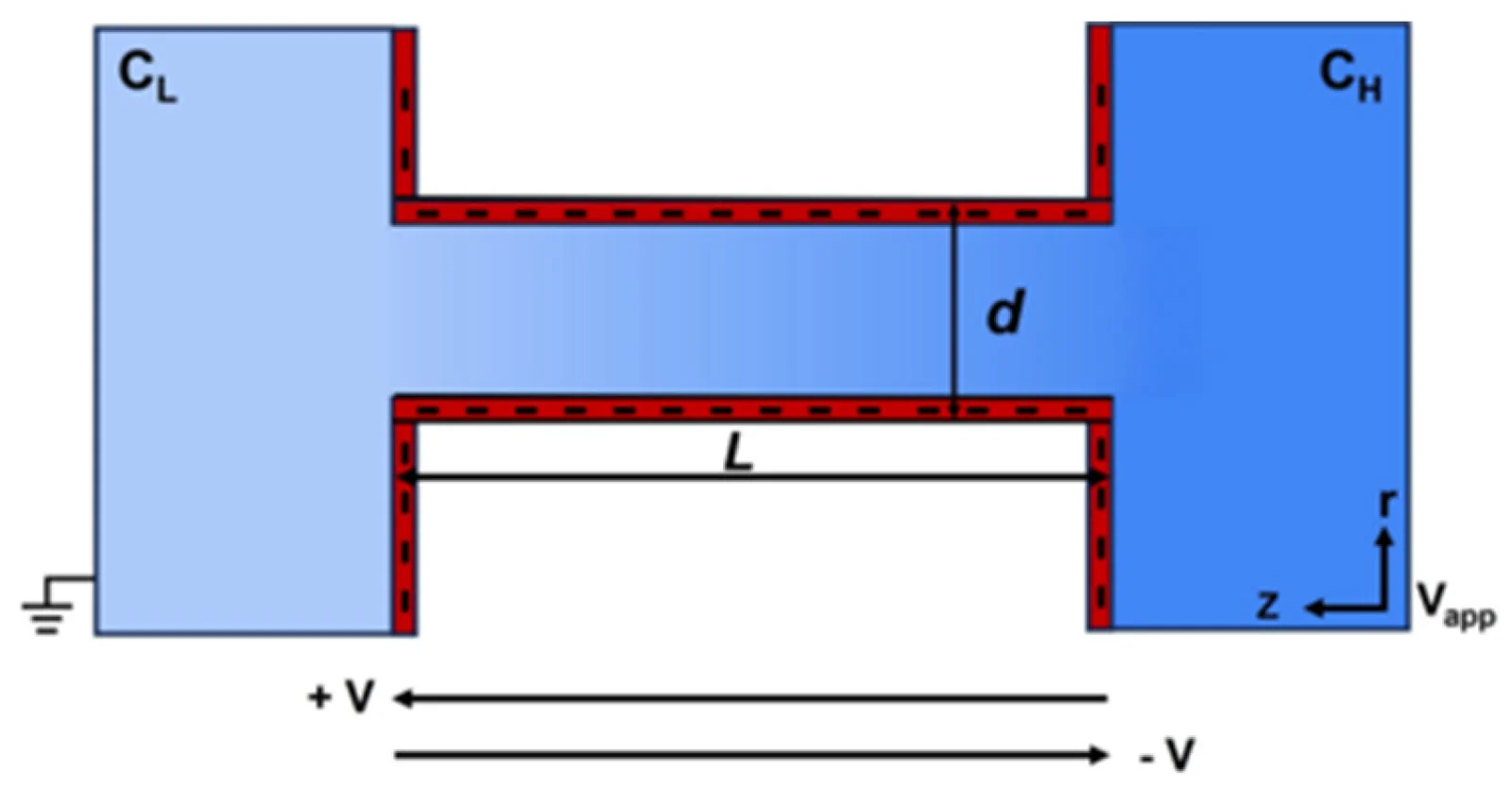
Schematic of the cylindrical macropore model. *C_L_* and *C_H_* represent the low salt concentration and high salt concentration, respectively.

A multiphysics coupling model is established using COMSOL Multiphysics 6.3 to perform numerical simulations of ion transport behavior within macropores. The computational model is shown in Figure 1 and consists of two reservoirs with different solution concentrations connected by a cylindrical macropore. The baseline model is configured with a macropore length of 12,000 nm, a pore diameter of 180 nm, a surface charge density of −0.02 C/m^2^, and a concentration ratio of 100 between the high− and low−concentration reservoirs. Su et al. [41] report that the predicted variation in ion rectification is generally consistent between the constant and pH−dependent surface charge models. This consistency suggests that the constant surface charge model reliably represents pore ion transport under certain pH and ionic conditions and is suitable as a boundary condition for studying ion transport mechanisms. On this basis, the numerical simulations in this study employ a constant surface charge boundary condition. The system temperature is set to 298 K, and the relative permittivity of water is set to 80. KCl solution is selected as the electrolyte, which is commonly used in macropore biosensing studies [42,43]. The concentration in the low−concentration reservoir is maintained at a constant value of 10 mM.

At 298 K, the Debye length of a KCl solution is expressed as λ_D_ = 0.304/$\sqrt{C}$ nm, where C denotes the ionic strength in mol/L(M) [44]. The Debye lengths in 10, 100, 200, 500, and 1000 mM KCl solutions are approximately 3.04, 0.96, 0.68, 0.43, and 0.304 nm, respectively. Therefore, even on the low−concentration side at 10 mM, where the Debye length is the largest, the minimum pore diameter of 180 nm used in this study is approximately 59 times the Debye length. Within the pore diameter range investigated in this study, the electrical double layers near the pore walls do not overlap significantly across the entire pore cross section, and the pore center retains approximately electroneutral bulk−like characteristics. Based on these characteristics, this study treats ions as continuous concentration fields and uses the Poisson−Nernst−Planck (PNP) equations to describe ionic diffusion, electromigration, and convection. The electrolyte solution is modeled as an incompressible Newtonian fluid, and the Navier−Stokes (NS) and continuity equations describe momentum and mass conservation, respectively. These equations are coupled to solve the ion transport, electric, and flow fields [45,46,47,48]. This continuum model is widely used to study electroosmotic flow, concentration polarization, and ion rectification in macropores [34,36,49,50].

(1) $${\varepsilon \nabla }^{2}\varphi =-\sum_{i=1}^{N}z_{i}FC_{i},$$

(2) $$\nabla \cdot J_{i}=\nabla \cdot \left(C_{i}v-D_{i}\nabla C_{i}-\frac{{Fz}_{i}C_{i}D_{i}}{RT}\nabla \varphi \right)=0,$$

(3) $$\mu \nabla^{2}v-\nabla p-\sum_{i=1}^{N}\left(z_{i}FC_{i}\right)\nabla \varphi =0,$$

(4) ∇⋅v=0,

where ε represents the dielectric constant of the electrolyte solution, *φ* is the electric potential, and *N* denotes the total number of ionic species in the solution. The parameters *z_i_*, *C_i_*, *J_i_*, and *D_i_* represent the valence number, molar concentration, molar flux vector, and diffusion coefficient of the i−th ion, respectively. *F* is the Faraday constant, *v* is the fluid velocity vector, *R* is the ideal gas constant, *T* is the absolute temperature of the system, *μ* is the dynamic viscosity of the electrolyte solution, and *p* is the fluid pressure.

The ionic current *I* is obtained by integrating the anion and cation fluxes over the reservoir boundary according to Equation (5) [51,52,53].

(5) $$I=\int_{S}F\left(\sum_{i=1}^{2}z_{i}J_{i}\right)\cdot n\;dS$$

where *I* denotes the total ionic current, *S* is the integration cross section, and *n* is the unit normal vector to *S*.

The effects of the concentration gradient, external electric field, surface charge density, and macropore structural parameters on the NDR effect are systematically investigated through numerical simulations. The concentration of the high−concentration reservoir is varied from 100 to 1000 mM, whereas the low−concentration reservoir remains constant at 10 mM, giving concentration ratios of 10–100. The macropore surface is assigned a negative charge, and the surface charge density range is determined from experimentally reported values for typical nanofluidic materials. The reported surface charge densities are approximately −0.005 C/m^2^ for Si_3_N_4_, −0.02 C/m^2^ for SiO_2_, and −0.08 C/m^2^ for polyethylene terephthalate (PET) membranes [54,55,56,57]. Furthermore, the surface charge density of these materials can be effectively controlled through solution pH adjustment or surface functionalization [58,59]. Accordingly, the macropore surface charge density is varied from −0.02 to −0.08 C/m^2^ within the experimentally accessible range to investigate ion transport behavior at different negative charge intensities. The macropore length and pore diameter are varied from 1000 to 18,000 nm and from 180 to 800 nm, respectively.

**Figure 2:**
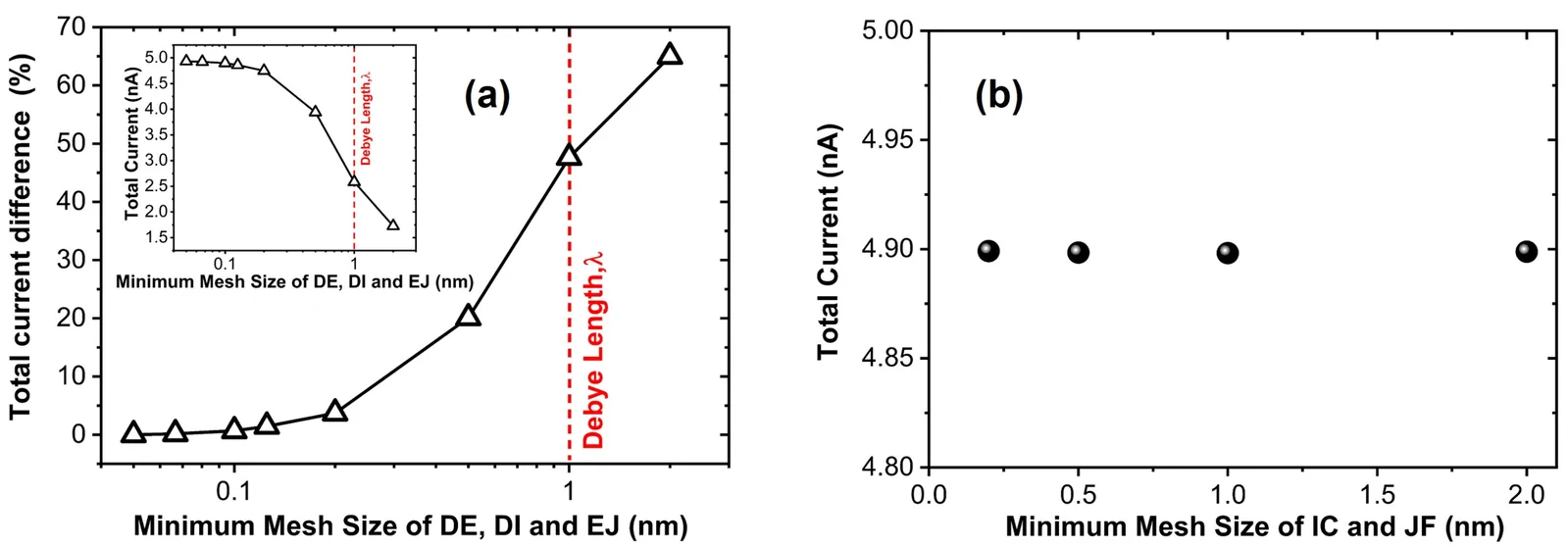
Effect of different mesh sizes on ionic current within the macropore. (**a**) Effect of different mesh sizes on ionic current in the DE, DI, and EJ regions; (**b**) effect of different mesh sizes on ionic current in the IC and JF regions.

The mesh generation scheme for the model is shown in the schematic diagram in Table 1. The regions within 3 μm of the macropore inner and outer surfaces are refined with a mesh size of 0.1 nm, while the remaining regions use a mesh size of 0.5 nm. Figure 2a shows the influence of different mesh sizes in the regions within 3 μm of the macropore inner surface and pore entrance (DE, DI, and EJ) on the ionic current. The ionic current obtained with the minimum mesh size of 0.05 nm is 4.9 nA and is used as the reference value. As the mesh size increases, the relative deviation of the ionic current first increases and then reaches a stable value. When the minimum mesh size is 0.1 nm, the relative deviation of the ionic current is only 0.68%, indicating that this mesh size provides sufficient accuracy for calculating ion and fluid transport within the electric double layer region. Figure 2b further evaluates the influence of mesh size in the other external regions (IC and JF) on the ionic current. The calculated ionic current remains stable as the mesh size varies in these regions, indicating that a minimum mesh size of 0.5 nm can satisfy the accuracy requirements of the simulations.

## 3. Results and Discussion

### 3.1. Formation Mechanism of Concentration Gradient−Induced NDR

**Figure 3:**
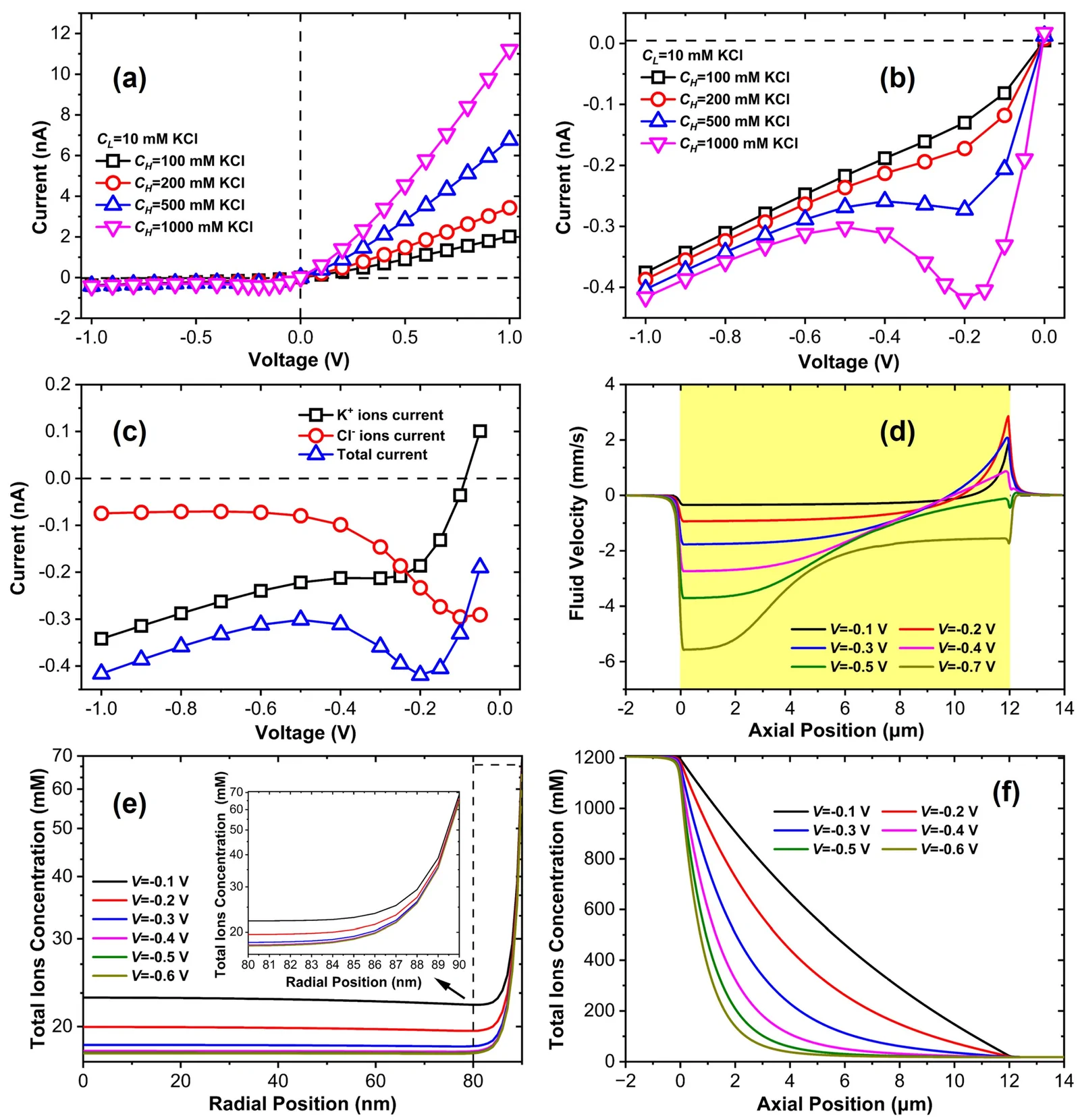
Mechanistic analysis of concentration gradient−induced NDR behavior in macropores. (**a**) I–V curves under different concentration gradients. (**b**) I–V curves in the negative voltage region under different concentration gradients. (**c**) Variations in total ionic current and K^+^/Cl^−^ ionic currents with applied voltage in the negative voltage region. (**d**) Axial distributions of fluid velocity at different voltages, where the yellow shaded region represents the macropore region. (**e**) Radial distributions of ion concentrations near the low−concentration side entrance. (**f**) Axial distributions of ion concentrations along the macropore. Panels (**c**–**f**) are obtained under a concentration gradient of C_H_:C_L_ = 1000 mM:10 mM.

Figure 3 presents the current−voltage (I–V) characteristics and current response in the negative voltage region under various concentration gradients. The macropore length and diameter were fixed at 12,000 nm and 180 nm, respectively. The KCl concentration on the low−concentration side was maintained at 10 mM, whereas that on the high−concentration side was varied from 100 to 1000 mM to establish a series of concentration gradients. The working electrode was consistently positioned on the high−concentration side. Figure 3a shows the macropore current as a function of voltage under different concentration gradients. Under positive voltage, the current increased with the voltage. At a concentration gradient of 10:100 mM, the current at 1 V was 2.02 nA, representing a 124.4% increase over the 0.90 nA obtained at 0.5 V; when the concentration on the high−concentration side was increased to 1000 mM, this increase reached 146.7%. Thus, the increase became more pronounced as the concentration gradient widened. Because the concentration on the low−concentration side was constant, the currents at negative voltages appear similar in Figure 3a; therefore, this region is magnified in Figure 3b. Across the different concentration gradients, the current exhibited a nonmonotonic trend under negative voltages−specifically, an NDR effect−characterized by an initial increase, followed by a decrease and then a further increase, as the magnitude of the negative voltage increased. This NDR characteristic became more distinct with an increasing concentration gradient. The NDR region, in which the current decreases as the voltage magnitude increases, was primarily located between −0.2 V and −0.5 V, with the current reaching a local maximum near −0.2 V in all cases.

Figure 3c shows the cation, anion, and total ionic currents as functions of voltage at a concentration ratio of 10 mM:1000 mM. The K^+^ ion current exhibits a rapid decline within the voltage range from 0 V to −0.2 V, while it slowly increases as the voltage further rises to −1 V. In contrast, the Cl^−^ ion current decreases rapidly when the voltage is below −0.5 V, but its rate of decline significantly reduces once the voltage exceeds −0.5 V. The combined effects of these two ionic currents cause the total current to decrease as the voltage magnitude increases within the −0.2 V to −0.5 V range, producing the NDR phenomenon. For K^+^ ions, concentration gradient−driven diffusion opposes electric field−driven electromigration. When the magnitude of the negative voltage is less than 0.05 V, corresponding to the range between 0 V and −0.05 V, the K^+^ current is positive, indicating that diffusion is dominant. As the voltage magnitude increases to 0.2 V, the K^+^ current changes from positive to negative, indicating that the electric field effect has become slightly stronger than the concentration gradient effect. Once the negative voltage exceeds −0.5 V, the electric field becomes dominant, and the current−voltage relationship returns to an approximately linear form. Conversely, for Cl^−^ ions, concentration gradient−driven diffusion and electric field driven migration are aligned, both proceeding from the high−concentration side to the low−concentration side. As the magnitude of the negative voltage increases, the concentration difference between the two sides decreases, causing a continuous decline in the Cl^−^ current. Within the −0.2 V to −0.5 V range, the electric field effect slightly outweighs the concentration gradient effect, and the rapid decay in the Cl^−^ current dominates the total current response. Consequently, the total current decreases as the voltage magnitude increases, giving rise to NDR.

Figure 3d shows the axial fluid−velocity distribution along the macropore axis under various applied negative voltages at a concentration ratio of 1000 mM:10 mM. An abrupt change in fluid velocity occurs at the inlet on the low−concentration side at all voltages, indicating the formation of a local vortex in the inlet region. The vortex velocity reaches a maximum at −0.2 V; this flow characteristic effectively retards the dilution of Cl^−^. Consequently, the Cl^−^ concentration remains relatively high near −0.2 V, producing a local maximum in the total current at this voltage. Figure 3e and Figure 3f show the total ion concentration distributions at the inlet on the low−concentration side and along the pore axis, respectively. When the negative voltage exceeds −0.2 V, the total ion concentration in the inlet region remains low, and further increases in voltage cause the ion concentration to level off. The concentration gradient drives ionic diffusion, while the applied electric field drives electromigration. Their combined effects govern ion transport within the pore and induce dynamic concentration polarization at the pore entrance. Once the local conductivity loss due to ion depletion exceeds the current increase caused by enhanced electromigration, the total current decreases as the applied voltage increases, leading to NDR behavior.

The NDR behavior obtained from the simulations is further compared with the corresponding experimental results. Rabinowitz et al. [21] observed NDR over the voltage range of 0.6–1.0 V at a solution concentration ratio of approximately 100 between the two sides. They attributed this phenomenon to concentration gradient−induced nonlinear electroosmotic flow and local ion depletion. Luo et al. [19] reported NDR between −0.85 and −0.86 V in a negatively charged conical glass nanopore. They identified the competition between pressure−driven and electroosmotic flows as the main cause. Lin et al. [30] reported that charged mesopores under pH regulation exhibit NDR mainly at applied voltages of 0.6–0.8 V. This effect mainly arises from the oppositely directed ionic diffusion and electroosmotic flow, which create local ion−depletion and low conductivity regions within the pore, thereby reducing the current. Previous experiments typically employed conical macropores and involved different electrolytes and boundary conditions. Therefore, the reported characteristic voltage windows are comparable to that of the present cylindrical macropore system only in terms of order of magnitude. The observed local vortices slow Cl^−^ dilution, while the entrance region remains at a low ion concentration. The close agreement with the experimental findings above strongly confirms the validity of the parameters adopted in this study and the resulting simulation results.

### 3.2. Regulation of NDR by Pore Length

**Figure 4:**
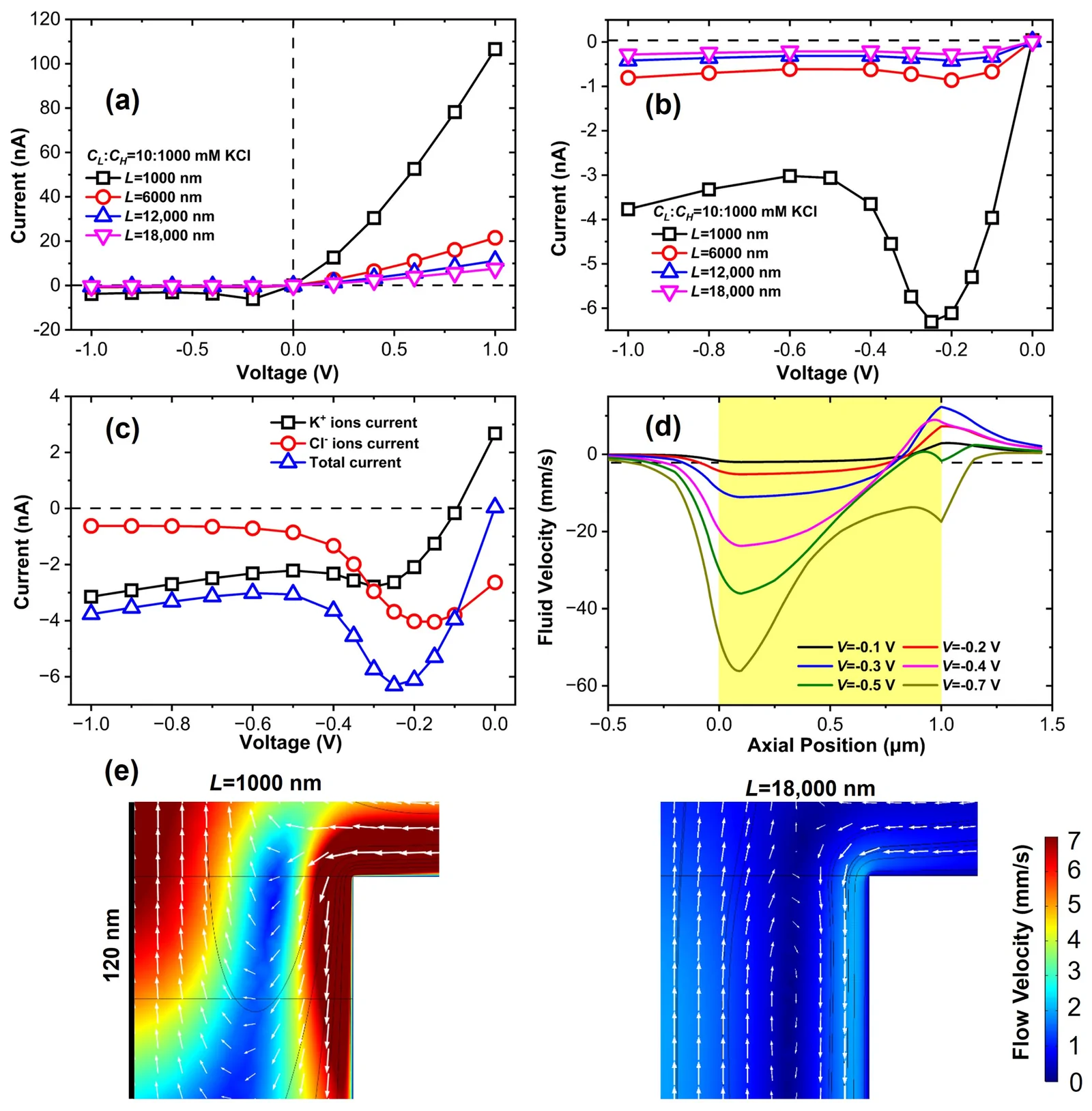
Effects of macropore length on NDR behavior and ionic transport characteristics. (**a**) I–V curves for macropores with different lengths. (**b**) I–V curves in the negative voltage region for different macropore lengths. (**c**) Total ionic current and K^+^/Cl^−^ ionic currents as a function of applied voltage in the negative voltage region. (**d**) Axial distributions of fluid velocity at different voltages, where the yellow shaded region represents the macropore region. (**e**) Velocity contours and streamlines near the low concentration side entrance of macropores with lengths of L = 1000 nm and L = 18,000 nm at V = −0.2 V. Panels (**c**,**d**) are obtained under the condition of L = 1000 nm.

To further investigate the effect of the pore length on the NDR, four systems with pore lengths of 1000 nm, 6000 nm, 12,000 nm, and 18,000 nm were compared while the concentration ratio was fixed at 1000 mM:10 mM and the pore diameter at 180 nm. Figure 4a,b present the I–V curves for different pore lengths. In all systems, the NDR appears exclusively in the negative voltage range, with its characteristic voltage window consistently located between −0.5 V and −0.2 V. As the pore length increases, the peak−to−valley characteristics of NDR diminish, indicating that shorter pores enhance the NDR behavior. Figure 4c shows the individual ionic current components as functions of voltage for L = 1000 nm. The K^+^ current changes rapidly at low voltages but tends to level off as the voltage increases further. The Cl^−^ current initially increases slightly with voltage, then decreases rapidly, and finally stabilizes. The superposition of these two currents produces a local maximum in the total current near −0.2 V. Between −0.2 V and −0.5 V, the total current decreases as the voltage increases, exhibiting NDR; this behavior is consistent with the preceding concentration gradient analysis. Figure 4d shows the fluid−velocity distribution along the axis of the 1000 nm macropore under various applied voltages. An abrupt change in fluid velocity occurs at the macropore opening on the low−concentration side. The fluid velocity reaches its maximum positive value at approximately −0.2 V. As the voltage magnitude increases to 0.5 V, corresponding to an applied voltage of −0.5 V, the velocity decreases to zero and then increases in the opposite direction. The vortex intensity in the inlet region peaks at approximately −0.2 V, effectively suppressing the decay of the Cl^−^ current and promoting formation of the total current peak at −0.2 V.

To further elucidate how the pore length regulates the NDR, Figure 4e presents the flow−velocity contours and streamline distributions within a 120 nm region at the inlet on the low−concentration side. For L = 1000 nm, pronounced axial and radial velocity gradients occur near the inlet, accompanied by a dense streamline distribution and clearly discernible recirculation structures. By contrast, the L = 18,000 nm system exhibits smaller flow−field gradients and a more linear streamline distribution. The pore length controls the distances of ionic migration and diffusion, as well as the penetration depth of the concentration polarization region from the pore entrance. Shorter pores enhance concentration polarization and local vortices near the entrance, causing more pronounced changes in the K^+^ and Cl^−^ current components and strengthening the peak−to−valley characteristics of NDR. Consequently, shorter pore lengths are more conducive to NDR.

### 3.3. Regulation of NDR by Pore Diameter

**Figure 5:**
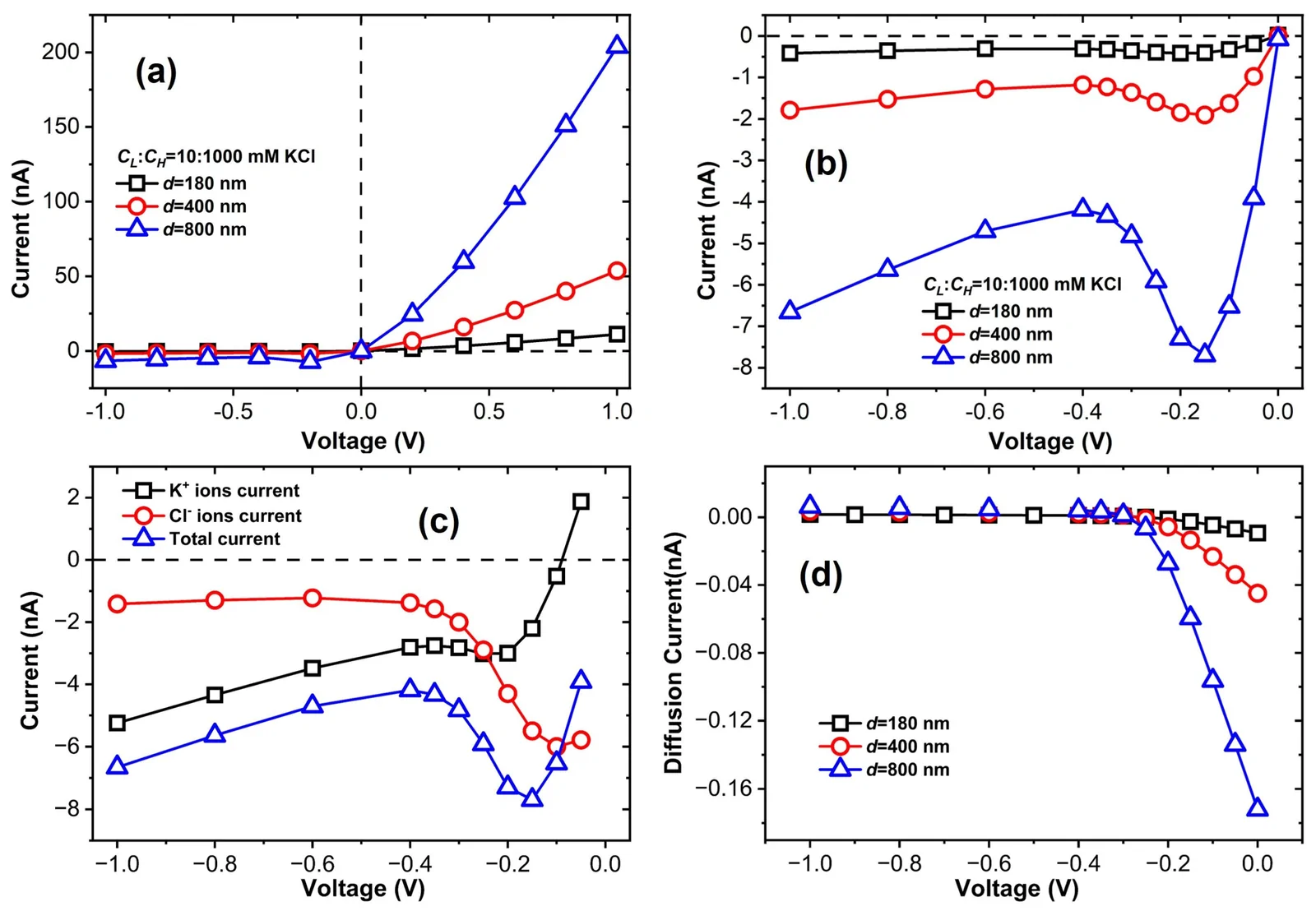
Effects of macropore diameter on ionic transport characteristics and NDR behavior. (**a**) I–V curves for macropores with different diameters. (**b**) I–V curves in the negative voltage region for different macropore diameters. (**c**) Applied voltage dependence of the total ionic current and K^+^/Cl^−^ ionic currents in the negative voltage region. (**d**) Diffusion currents driven by the concentration gradient as a function of the applied voltage under different macropore diameters.

To investigate the effect of the pore diameter on the NDR, systems with pore diameters of 180, 400, and 800 nm were analyzed at a concentration ratio of 1000 mM:10 mM and a pore length of 12,000 nm. The pore diameter predominantly affects the relative extent of the electrical double layer in the pore, the effective ion transport area, and the local electric field strength [60]. At smaller pore diameters, ion selectivity increases, and concentration polarization near the pore entrance becomes more pronounced. Therefore, variations in pore diameter alter the balance between ion depletion and electromigration compensation, thereby regulating the onset voltage and current reduction of NDR. Figure 5a,b present the I–V curves for these systems. In all cases, the NDR occurs exclusively within the negative voltage range from −0.2 V to −0.5 V. The NDR effect is significantly stronger for the 800 nm pore than for the other two pores, indicating that increasing pore diameter enhances the NDR. Figure 5c shows the individual ionic current components as functions of applied voltage for the 800 nm pore. As the voltage changes from 0 V to −1 V, the K^+^ current initially decreases rapidly, changing from positive to negative, and then slowly increases in the opposite direction. Meanwhile, the Cl^−^ current increases sharply, peaks near −0.1 V, and then gradually decreases as the voltage becomes more negative. The combined effects of these two ionic currents produce a local maximum in the total current near −0.2 V. Between −0.5 V and −0.2 V, the total current decreases as the voltage increases, exhibiting characteristic NDR.

Figure 5d compares the concentration gradient−driven diffusion current as a function of voltage for different pore diameters. In the low−voltage range between 0 and −0.3 V, the 800 nm pore exhibits the highest diffusion current, indicating a substantially higher diffusion−supply capability than the other two pores. Once the voltage exceeds −0.3 V, the diffusion currents for all three pore sizes converge, indicating no significant difference in diffusion capability. This comparison shows that larger macropores have a stronger advantage in concentration gradient−driven transport in the low−voltage regime. Because this enhanced diffusion supply competes effectively with the electric field, the system reaches a higher current peak at −0.2 V. Although its diffusion capability becomes comparable to those of the other pore sizes in the −0.2 V to −0.5 V range, the higher initial peak produces a more pronounced current decay; consequently, NDR is more distinct in the 800 nm system.

### 3.4. Regulation of NDR by Surface Charge Density

**Figure 6:**
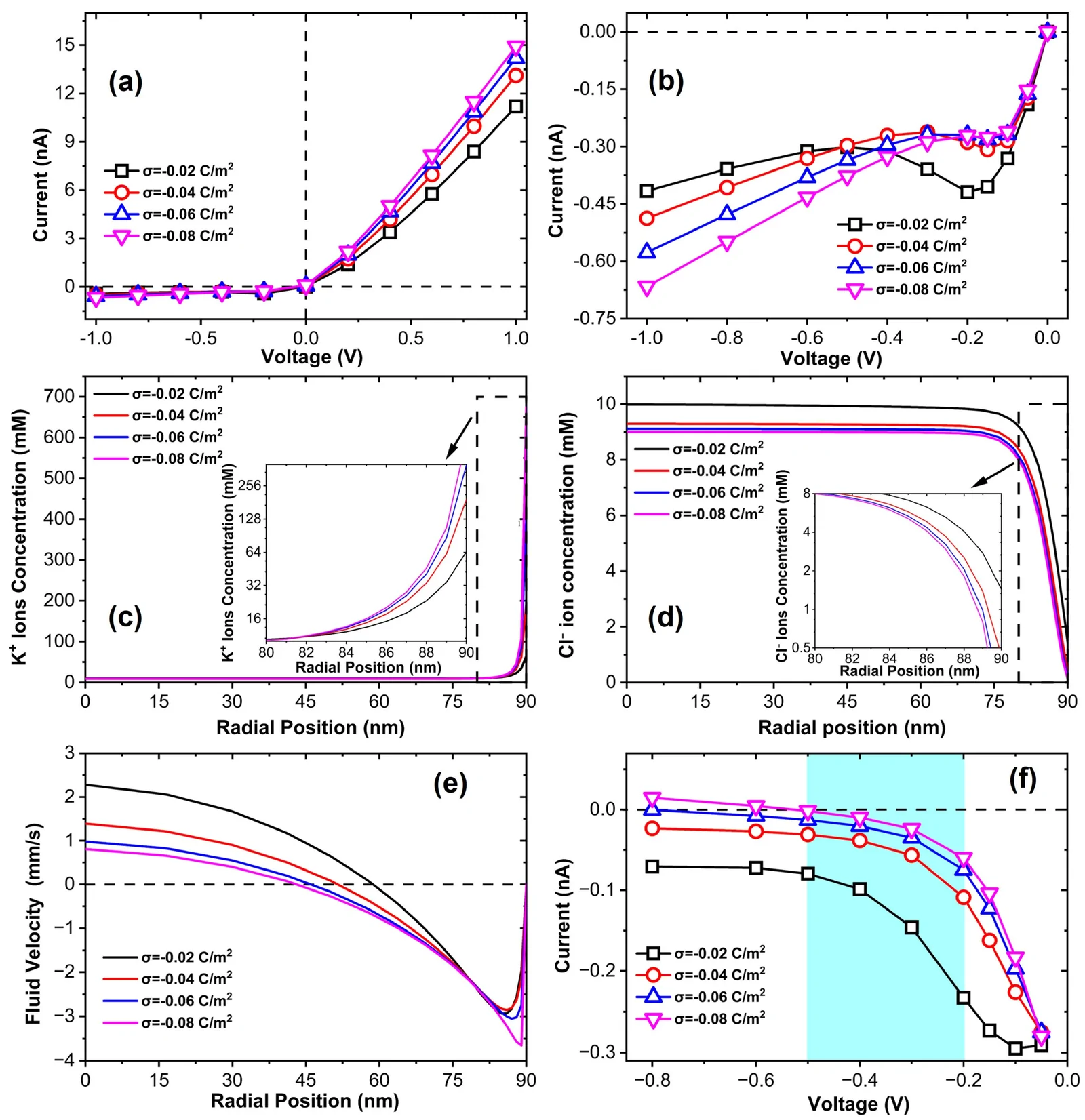
Effects of surface charge density on ionic transport characteristics and NDR behavior in macropores. (**a**) I–V curves of macropore systems with different surface charge densities. (**b**) I–V curves in the negative voltage region under different surface charge densities. (**c**) Radial concentration distributions of K^+^ ions near the low−concentration side entrance. (**d**) Radial concentration distributions of Cl^−^ ions near the low−concentration side entrance. (**e**) Radial distributions of fluid velocity near the low−concentration side entrance. (**f**) Variation in Cl^−^ ion current with applied voltage in the negative voltage region, where the light blue shaded region indicates the characteristic NDR voltage range from −0.5 V to −0.2 V. The applied voltage in panels (**c**–**e**) is −0.2 V.

To investigate how the surface charge density modulates the NDR effect, surface charge densities of −0.02 C/m^2^, −0.04 C/m^2^, −0.06 C/m^2^, and −0.08 C/m^2^ were selected, with a concentration ratio of 1000 mM:10 mM, a pore length of 12,000 nm, and a pore diameter of 180 nm. Figure 6 illustrates the current response and the distribution characteristics in the region near the pore entrance under these varying surface charge density conditions. Figure 6a,b present the I–V curves for the different systems. NDR occurs only between −0.5 V and −0.2 V; however, a distinct peak−to−valley characteristic is observed only at σ = −0.02 C/m^2^, indicating that lower surface charge densities favor NDR. Figure 6c shows the radial K^+^ concentration distribution at the inlet on the low−concentration side at V = −0.2 V. Near the wall, the K^+^ concentration increases with the surface charge density, strengthening the wall confinement of the cations and reducing effective K^+^ transport in the macropore bulk. Figure 6d shows the radial Cl^−^ concentration distribution under the same conditions. Near the wall, the Cl^−^ concentration decreases as the surface charge density increases, reducing the total amount of Cl^−^ contributing to the current response. Consequently, the Cl^−^ current is less likely to form a distinct peak near −0.2 V, weakening the NDR.

Figure 6e displays the radial distribution of fluid velocity at the inlet on the low−concentration side when V = −0.2 V. At σ = −0.02 C/m^2^, the flow velocity is highest at the pore center and relatively low near the walls. This velocity profile enhances the electroosmotic flow intensity, allowing the electric field to dominate over the ion diffusion driven by the concentration gradient. Higher surface charge densities attenuate the flow field gradient in the inlet region, thereby reducing the relative strength of the electric field. Figure 6f shows the variation in the Cl^−^ current with voltage for different surface charge densities. The light blue shaded region indicates the characteristic voltage window for NDR (−0.5 V to −0.2 V) determined from the total I–V curves−highlighting the contribution of Cl^−^ current decay to the drop in total current within this range. It should be noted that this shading merely marks a consistent voltage interval and does not imply that equally pronounced NDR peak−valley characteristics appear under all surface charge density conditions. At any given voltage, the Cl^−^ current is highest for σ = −0.02 C/m^2^ and lowest for σ = −0.08 C/m^2^; the larger Cl^−^ current provides a substantial basis for charge transport underlying the NDR phenomenon. Within the −0.2 V to −0.5 V range, the decay of the Cl^−^ current is most pronounced at σ = −0.02 C/m^2^, whereas the changes are relatively gradual for the other three surface charge densities. The rapid decay in the Cl^−^ current is the direct cause of the NDR phenomenon; consequently, lower surface charge densities facilitate the maintenance of diffusion−driven Cl^−^ supply channels, resulting in a more significant NDR effect. An increase in negative surface charge density strengthens the electrostatic attraction of the pore walls toward K^+^ and their repulsion of Cl^−^, which restricts Cl^−^ diffusion to the low−concentration side. By contrast, a lower surface charge density maintains sufficient Cl^−^ transport, leading to a higher initial current and a larger current reduction during ion depletion, thereby producing a more pronounced NDR [38].

## 4. Conclusions

This study employs a coupled Poisson−Nernst−Planck (PNP) and Navier−Stokes (NS) model to systematically investigate how the concentration gradient, applied electric field, pore parameters, and surface charge density regulate NDR behavior in cylindrical macropores. Because the pore diameters considered are substantially larger than the Debye length of the KCl solution, the electrical double layers near the pore walls do not significantly overlap across the entire pore cross section. Therefore, the primary mechanism underlying NDR is the competition between concentration gradient−driven diffusion and electric field−driven electromigration. Within the characteristic voltage range of −0.5 to −0.2 V, electromigration is stronger than concentration gradient−driven diffusion for Cl^−^ ions, whereas its effect on K^+^ ion transport is relatively weak. The Cl^−^ ion current decreases rapidly and dominates the reduction in the total ionic current, which leads to the NDR effect. The concentration gradient, geometric parameters of the macropore, and surface charge density work together to regulate the NDR response. A larger concentration gradient enhances the NDR response. The total ionic current reaches a local extremum near −0.2 V at all concentration gradients. A shorter pore length strengthens concentration polarization and local vortex effects near the low−concentration side entrance, which suppress the decrease in Cl^−^ ion current and enhances the NDR behavior. Increasing the pore diameter enhances ion diffusion transport and enables the system to achieve a higher current peak near −0.2 V. Decreasing the surface charge density reduces cation accumulation near the pore walls and facilitates continuous Cl^−^ ion diffusion through the nanochannel. A larger pore diameter, shorter pore length, weaker surface charge density, and increased concentration gradient are favorable for enhancing the NDR characteristics by increasing the Cl^−^ ion current. This study clarifies the effects of the concentration gradient, pore geometry, and surface charge density on ion transport and NDR behavior. It provides a theoretical foundation for optimizing ionic selectivity and transport resistance in charged nanopore/macropore devices and proposes a new design strategy for improving biosensing performance using salinity gradients.

## Figures and Tables

**Table 1:** Boundary conditions of the simulation model.

Model Schematic	Boundary	Poisson Equation	N−P Equation	N−S Equation
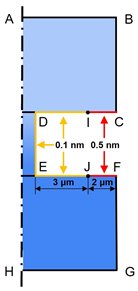	AB	Fixed potential *ϕ* = 0 (ground)	Fixed concentration *c_i_* = *C_L_*	Fixed pressure *p* = 0 Zero viscous stress n∙[*µ*(∇u + (∇u)^T^)] = 0
	BC, FG	Zero surface charge −**n**∙(*ε*∇*ϕ*) = 0	No flux **n**∙**N***_i_* = 0	No slip u = 0
	DC, EF DE	−**n**∙(*ε*∇*φ*) *= σ_w_*	No flux **n**∙**N***_i_* = 0	No slip u = 0
	HG	Fixed potential *ϕ* = *V_app_*	Fixed concentration *c_i_* = *C_H_*	Fixed pressure *p* = 0 Zero viscous stress **n**∙[*µ*(∇**u** + (∇**u**)^T^)] = 0
	AH	Axisymmetry	Axisymmetry	Axisymmetry

Note: *φ*, *V_app_*, *ε*, *C_L_*, *C_H_*, *p*, *n*, *N_i_*, *u*, *σ_w_*, and *μ* denote the electric potential, applied voltage, permittivity, bulk concentrations in the low− and high−concentration reservoirs, pressure, unit normal vector, ionic flux, fluid velocity, surface charge density, and dynamic viscosity of the solution, respectively.

## Data Availability

The data that support the findings of this study are available from the corresponding author upon reasonable request.

## Abbreviations

The following abbreviations are used in this manuscript:

NDR	Negative Differential Resistance
PNP	Poisson−Nernst−Planck
NS	Navier−Stokes
I–V	Current−Voltage
*C_L_*	Low Salt Concentration
*C_H_*	High Salt Concentration
